# Alterations in fecal β-defensin-3 secretion as a marker of instability of the gut microbiota

**DOI:** 10.1080/19490976.2023.2233679

**Published:** 2023-07-18

**Authors:** Zarwa Saqib, Giada De Palma, Jun Lu, Michael Surette, Premysl Bercik, Stephen Michael Collins

**Affiliations:** Farncombe Family Digestive Health Research Institute, Department of Medicine, Faculty of Health Sciences, McMaster University, Hamilton, ON, Canada

**Keywords:** Irritable bowel syndrome, antimicrobials, high fat diet, high salt diet, stress, microbiota, defensins

## Abstract

Compositional changes in the microbiota (dysbiosis) may be a basis for Irritable Bowel Syndrome (IBS), but biomarkers are currently unavailable to direct microbiota-directed therapy. We therefore examined whether changes in fecal β-defensin could be a marker of dysbiosis in a murine model. Experimental dysbiosis was induced using four interventions relevant to IBS: a mix of antimicrobials, westernized diets (high-fat/high-sugar and high salt diets), or mild restraint stress. Fecal mouse β-defensin-3 and 16S rRNA-based microbiome profiles were assessed at baseline and during and following these interventions. Each intervention, except for mild restraint stress, altered compositional and diversity profiles of the microbiota. Exposure to antimicrobials or a high-fat/high-sugar diet, but not mild restraint stress, resulted in decreased fecal β-defensin-3 compared to baseline. In contrast, exposure to the high salt diet increased β-defensin-3 compared to baseline. Mice exposed to the mix of antimicrobials showed the largest compositional changes and the most significant correlations between β-defensin-3 levels and bacterial diversity. The high salt diet was also associated with significant correlations between changes in β-defensin-3 and bacterial diversity, and this was not accompanied by discernible inflammatory changes in the host. Thus, dietary change or antimicrobial exposure, both recognized factors in IBS exacerbations, induced marked dysbiosis that was accompanied by changes in fecal β-defensin-3 levels. We propose that serial monitoring of fecal β-defensins may serve as a marker of dysbiosis and help identify those IBS patients who may benefit from microbiota-directed therapeutic interventions.

## Introduction

The term dysbiosis is used here to describe changes in the microbiota composition, function or temporal stability and is thought to contribute to functional and inflammatory conditions of the gut. The gut microbial composition is influenced by both host factors including genetics, psychological stress, immune activation and altered physiology, and environmental factors such as infection, diet and antibiotic exposure.^1–3^ Each of these factors has been implicated in the development or exacerbation of IBS. Enteric infection or extensive antibiotic exposure is the recognized risk factor for the development of IBS,^4,5^ and diet is a common trigger of symptoms in IBS.^6–8^ These observations rationalize the choice of interventions such as antibiotic exposure, dietary change, and stress to induce dysbiosis in the present study. Although, the intestinal microbiota is stable in healthy adult humans,^9,10^ the intra-subject variation in microbiota composition is less than that seen between individuals.^11–14^ Moreover, temporal instability of the human microbiota has been documented in some IBS patients.^15–17^ It follows that a simple marker of dysbiosis that reflects compositional instability of gut microbiota^18^ would be useful in identifying those IBS patients who might benefit from microbiota-directed therapies. Interestingly, a recent study^19^ showed that colonization of germ-free mice with IBS microbiota induced an IBS-like phenotype in the recipient mice, strongly implying that the microbiota contributes to the expression of IBS, and this was accompanied by an increase in β-defensin-3.

Defensins are small cationic antimicrobial peptides that play a role in maintaining homeostasis between the host and its resident microbes and protecting against pathogens.^20^ Defensins are produced mainly by colonic epithelial cells, Paneth cells and immunocytes in response to inflammatory stimuli or exogenous microbial substances,^21,22^ and serve as an effective barrier against the entry of pathogens into the host mucosa.^23–25^ Alpha and some β-defensins are constitutively expressed within the GI tract and most remain stable during inflammation.^26,27^ However, others like β-defensin-3 (a murine homologue of human β-defensin-2) are induced primarily upon pathogen encounter.^20,28,29^ Interestingly, defensins and other antimicrobial peptides also influence microbiota composition. For example, dysfunctional Paneth cells result in significant changes in microbiota composition.^30–32^

Changes in defensin secretion have been identified in IBS. Langhorst et al.^33^ revealed increased levels of human β-defensin-2 in the feces of IBS patients in the absence of macroscopic inflammation, as levels of fecal calprotectin and lactoferrin did not differ between healthy controls and patients with IBS. Similarly, increased concentrations of fecal β-defensin-2 were found in children with IBS, associated with abdominal pain and increased gut permeability, indicating activation of the innate immune system.^34^ However, decreased levels of β-defensin-2 have also been reported in patients with IBS compared to controls.^35^ A very recent study proposed that measurement of human β-defensin-2 levels, together with the inclusion of microbiome profiles, can serve as a marker to differentiate between IBD and IBS patients.^36^ Recently, we have shown that colonizing germ-free mice with IBS-D microbiota results in altered gut function, including faster gastrointestinal transit, altered gut permeability and secretion, which was accompanied by an increase in colonic β-defensin-3 expression in recipient mice.^19^ Together, these observations prompted the current proof-of-concept study in mice to determine whether the experimental perturbation of the intestinal microbiota, using interventions relevant to the natural history of IBS, will result in changes in fecal β-defensin-3 levels.

## Results

### Antimicrobials disrupt gut microbiota composition and decrease fecal β-defensin secretion in mice

**Figure 1.**
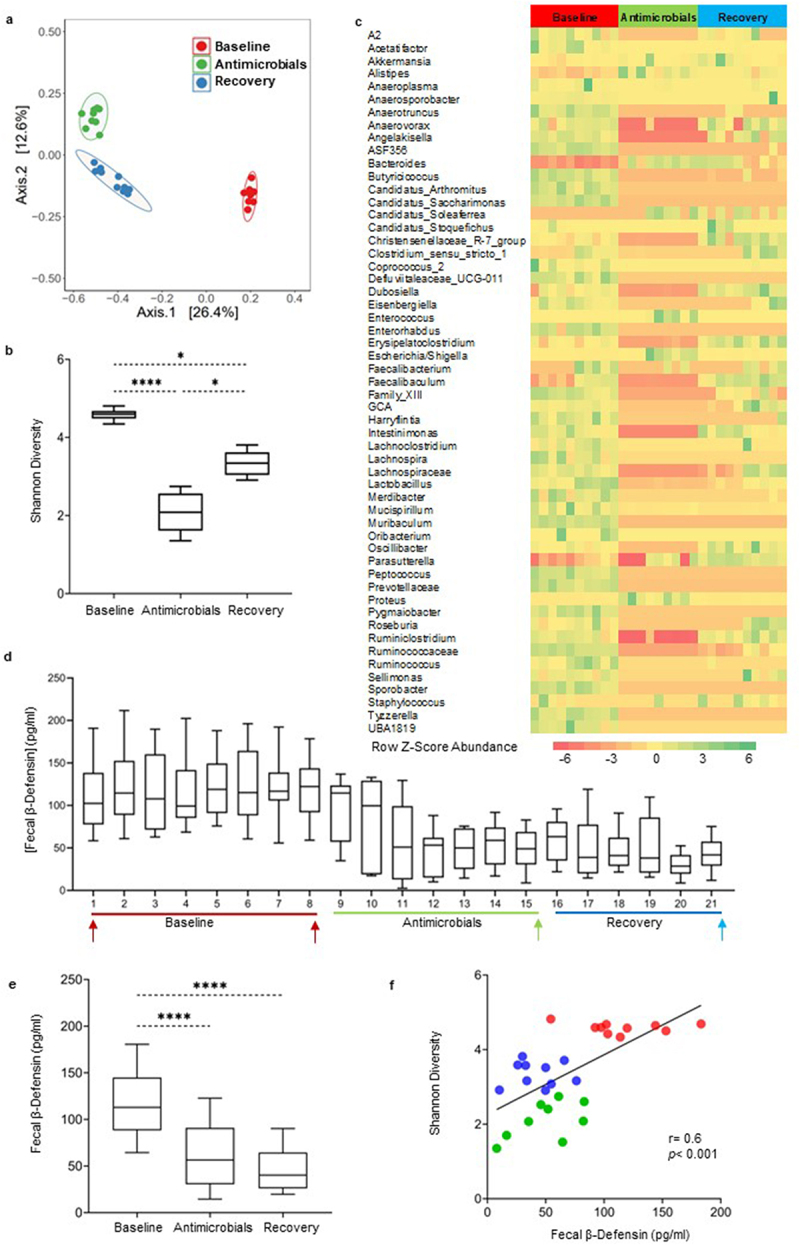
The antimicrobial cocktail alters gut microbiota composition and induces changes in fecal β-defensin secretion in mice. a) PCoA plot based on a Bray Curtis distance matrix analysis of the fecal microbiota composition over the course of the experiment in mice. Community composition was compared by ADONIS2 with 999 permutations. Each dot represents the bacterial microbiota of an individual mouse. Ellipses indicate 95% confidence intervals and were generated as a distribution around a centroid for each group based on ADONIS2. b) Changes in alpha-diversity using Shannon diversity index, analysis was performed by 1- way ANOVA followed by Dunn’s testing for multiple comparisons. Data is presented as box and whisker plot with whiskers extending from 10^th^ to 90^th^ percentile. c) Heatmap of all the differentially abundant taxa (aggregated at genus level) in mice evaluated using ANCOM-BC. Statistical significance on differentially abundant taxa between groups was corrected for multiple comparisons using FDR (*q* < 0.05). Color intensity on heatmaps was generated by using z-scores calculated based on relative abundance of each bacterial taxa. d) Time-course graph showing fluctuations in fecal β-defensin-3 values in mice over the course of the experiment. Time points chosen for microbiota analysis are indicated with arrows. e) Composite of mean changes in β-defensin secretion during the three experimental timepoints, presented as a box and whisker plot with whiskers extending from 10^th^ to 90^th^ percentile. Statistical differences were calculated using the Kruskal–Wallis test followed by post hoc Dunn’s test for multiple comparisons. f) Correlation between changes in alpha diversity values and defensin secretion in mice during the course of the experiment. Red symbols represent associations between baseline and β-defensin-3 levels, while green and blue symbols represent intervention timepoints and day 21 of recovery time point, respectively. Correlations were generated using Spearman rank index. Baseline days 1 and 7 were combined for the analysis. *n* = 4–5 mice/group. *p* < 0.05 was considered significant. **** = *p* ≤0.0001, *** = *p* ≤0.001, ** = *p* ≤0.01, * = *p* ≤0.05.

A previously described mix of non-absorbable antimicrobials (AMC)^37,38^ caused a marked and persistent disruption of microbiota composition, as illustrated in the PCoA plot created using the Bray Curtis and Aitchison distance matrices (Figure 1a, Table 1, Supplementary Table S1). The stool microbiota profiles of the mice at baseline clustered together with a major drift following the AMC intervention and minimal reversal to baseline during the recovery period as shown in Figure 1a. Fecal microbial diversity did not revert to baseline one-week post-AMC as shown by the Shannon Diversity Index (Figure 1b). Alpha diversity was lower during the intervention period as compared to the baseline. No statistically significant changes in net bacterial load were found in mice (Supplementary Figure S2a and S2b). To confirm the impact of AMC on net microbial composition, we performed ANCOM-BC analysis to find differentially expressed bacteria. We found altered relative abundance of several bacteria taxa that persisted during recovery, and these included the genera *Anaerotruncus*, *Butyricicoccus*, *Candidatus Arthromitus, Candidatus Saccharimonas*, some species of *Clostridium*, *Defluviitaleaceae, Enterorhabdus, Lachnospira*, *Mucispirillum*, *Muribaculum*, *Peptococcus, Prevotellaceae*, *Sporobacter*, and *Tyzzerella*, among others (Figure 1c). While most of these bacteria were decreased during AMC administration, the abundance of the genera *Bacteroides* and *Proteus* increased in mice. AMC administration was accompanied by a decrease in β-defensin-3 levels (Figure 1d), which persisted during the recovery period (Figure 1e). There was a positive correlation between β-defensin-3 levels and overall bacterial diversity (Figure 1f), suggesting a linkage between these responses. Although no major differences were observed in the microbial composition and β-defensin-3 levels between males and females, subtle differences were noted. For example, while the stool microbiota profiles and bacterial taxa of all the males and females followed similar trends at baseline and during the intervention and recovery periods (Supplementary Figure S1a), only *Enterococcus* spp., *Escherichia* spp., and *Shigella* were increased in females during the intervention (Supplementary Figure S4). In addition, while a three-fold reduction in β-defensin-3 levels from baseline was observed in males, only over a one-fold decrease was seen in females during the AMC intervention (Supplementary Figure S1b).

**Table 1.** P and R^2^ (group variance) values based on the ADONIS2 statistical analysis of the Bray Curtis-distance matrix (top) and Aitchison distance matrix (bottom) on the effect of antimicrobials, high-fat/high-sugar diet and high salt diet on fecal microbiota composition in mice.

	AMC		HFHSD		HSD	
	p	R2	p	R2	p	R2
			**Bray-Curtis Distance Matrix**			
Baseline vs. Treatment	0.00	0.57	0.00	0.52	0.00	0.29
Treatment vs. Recovery	0.00	0.37	0.00	0.42	0.00	0.31
Baseline vs. Recovery	0.00	0.56	0.02	0.14	0.04	0.11
			**Aitchison Distance Matrix**			
Baseline vs. Treatment	0.00	0.54	0.00	0.34	0.00	0.19
Treatment vs. Recovery	0.00	0.37	0.00	0.27	0.00	0.21
Baseline vs. Recovery	0.00	0.45	0.00	0.14	0.00	0.10

We next explored the relationships between bacterial taxa that were found to be differentially abundant based on ANCOM-BC analysis (Figure 1c), β-defensin-3 levels and the Shannon diversity index. We identified several genera that correlated with not only β-defensin-3 levels but also Shannon diversity (Table 2). These included *Acetatifactor, Alistipes, Anaerotruncus*, *Anaerosporobacter, Bacteroides, Butyricicoccus, Candidatus Arthromitus, Candidatus_Saccharimona, Defluviitaleaceae, Enterorhabdus, Prevotellaceae, Lachnospira, Lactobacillus, Mucispirillium*, *Peptococcus*, and *Sporobacter*. Interestingly, bacteria possessing protective/anti-inflammatory properties correlated negatively with β-defensin-3 levels, suggesting that low β-defensin-3 may be due to the abundance of these counter-inflammatory bacteria such as *Bacteroides* (*r* = −0.6, *p* < 0.001) and *Alistipes* (*r* = −0.5, *p* = 0.01),^39,40^ as shown in Table 2. Conversely, the abundance of bacteria with pro-inflammatory properties correlated positively with low defensin levels (Table 2). For example, *Prevotellaceae* with reported pro-inflammatory properties^41,42^ (*r* = 0.6, *p* < 0.001), *Candidatus Arthromitus*^43^ (*r* = 0.8, *p* < 0.001), *Eisenbergiella*^44^ (*r* = 0.5, *p* = 0.01), and *Muribaculum* taxa^45^ (*r* = 0.6, *p* < 0.001) were all positively correlated with low defensin levels following AMC exposure.

**Table 2.** Correlations between the abundances of differentially abundant bacterial taxa (genus), defensin levels and microbial diversity (Shannon Diversity). Bacterial taxa that were found to be differentially abundant in ANCOM-BC analysis were correlated with β-defensin levels for all experimental conditions. Additional correlations were made to see whether the differentially abundant taxa that correlate with β-defensins also correlate with microbial diversity (Shannon index values were used). All correlations were based on relative abundances and were generated using the Spearman rank index followed by FDR corrections. *p* < 0.05 was considered significant following FDR correction.

Bacteria	β-Defensin-3		Shannon Diversity	
	r	p_adjust	r	p_adjust
	**AMC**			
Acetatifactor	0.64	.00	0.76	.00
Alistipes	−0.54	.01	−0.79	.00
Anaerosporobacter	0.61	.00	0.75	.00
Anaerotruncus	0.72	.00	0.81	.00
ASF356	0.69	.00	0.83	.00
Bacteroides	−0.58	.00	−0.78	.00
Butyricicoccus	0.67	.00	0.86	.00
Candidatus_Arthromitus	0.76	.00	0.82	.00
Candidatus_Saccharimonas	0.74	.00	0.81	.00
Defluviitaleaceae_UCG.011	0.77	.00	0.78	.00
Eisenbergiella	0.54	.01	0.78	.00
Enterococcus	−0.42	.04	-	-
Enterorhabdus	0.65	.00	0.75	.00
Family_XIII	0.57	.00	0.83	.00
Harryflintia	0.74	.00	0.83	.00
Lachnoclostridium	0.46	.03	0.61	.00
Lachnospira	0.75	.00	0.70	.00
Lactobacillus	0.58	.00	0.88	.00
Merdibacter	0.69	.00	0.65	.00
Mucispirillum	0.69	.00	0.73	.00
Muribaculum	0.60	.00	0.66	.00
Oribacterium	0.43	.03	-	-
Peptococcus	0.74	.00	0.82	.00
Prevotellaceae	0.59	.00	0.59	.00
Pygmaiobacter	0.72	.00	0.83	.00
Ruminococcaceae	0.59	.00	0.93	.00
Ruminococcus	0.63	.00	0.78	.00
Sporobacter	0.76	.00	0.81	.00
Tyzzerella	0.71	.00	0.83	.00
UBA1819	0.66	.00	0.81	.00
	**HFHSD**			
Anaerosporobacter	0.58	.00	0.77	.00
Anaerotruncus	−0.47	.03	-	-
Candidatus_Arthromitus	0.47	.04	0.56	.01
Eisenbergiella	0.60	.00	0.86	.00
Lachnoclostridium	0.49	.02	0.72	.00
Lactococcus	−0.49	.02	−0.45	.04
Parasutterella	0.48	.03	-	-
	**HSD**			
A2	0.46	.04	0.63	.00
Anaerotruncus	0.71	.00	0.70	.00
Blautia	0.46	.04	-	-
Butyricicoccus	0.56	.01	0.79	.00
Candidatus_Arthromitus	−0.70	.00	-	-
Clostridium_sensu_stricto_1	−0.46	.04	-	-
Eisenbergiella	0.50	.02	0.64	.00
Erysipelatoclostridium	0.61	.00	-	-
Harryflintia	0.64	.00	0.78	.00
Lactobacillus	−0.67	.00	−0.58	.01
Muribaculum	−0.51	.02	−0.60	.00
Oribacterium	0.46	.04	0.48	.03
Oscillibacter	0.55	.01	0.56	.01
Parabacteroides	0.68	.00	-	-
Ruminiclostridium	0.48	.03	0.83	.00
Ruminococcaceae	0.69	.00	-	-
Staphylococcus	0.75	.00	-	-

### High-fat/high-sugar diet transiently alters gut microbiota composition and decreases fecal β-defensin secretion in mice

**Figure 2.**
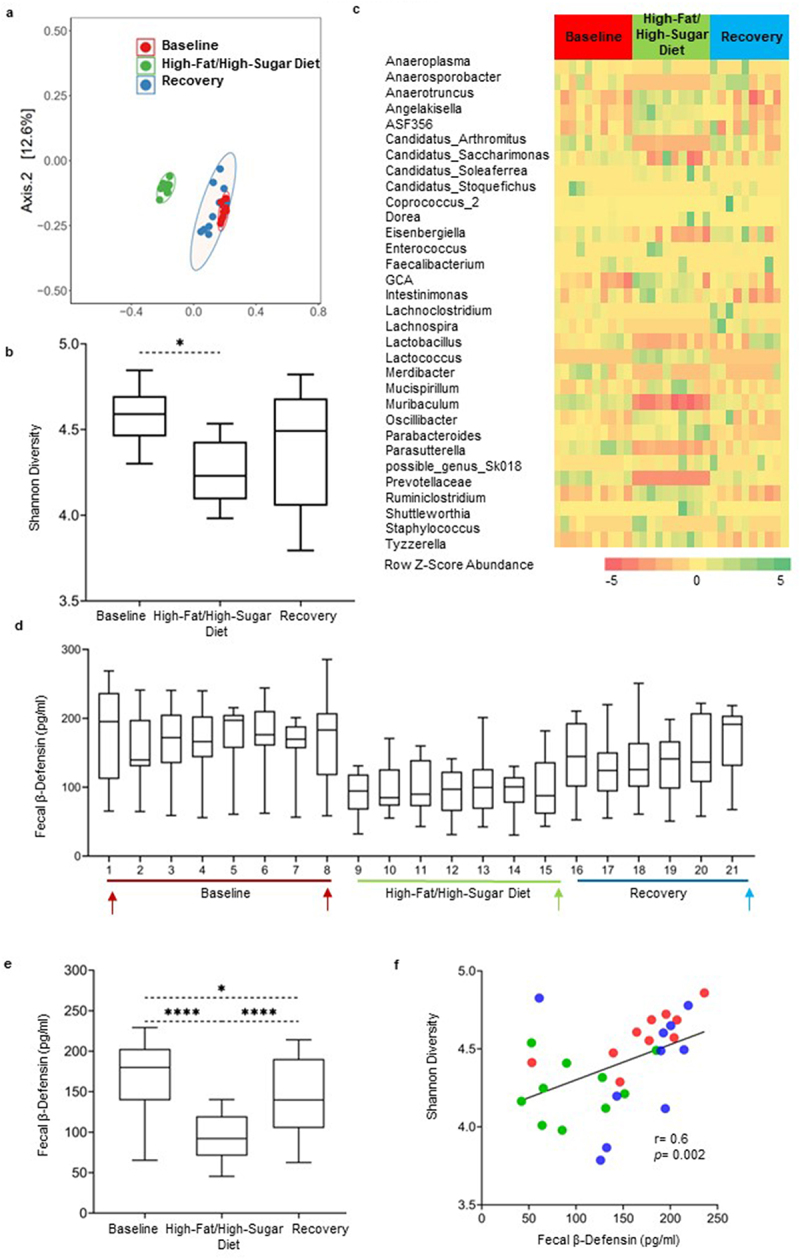
The high-fat/high-sugar diet administration transiently disrupts gut microbiota composition and alters fecal β-defensin secretion in mice. a) PCoA plot based on a Bray Curtis distance matrix analysis of the fecal microbiota composition over the course of the experiment in mice. Community composition was compared by ADONIS2 with 999 permutations. Ellipses indicate 95% confidence intervals and were generated as a distribution around a centroid for each group based on ADONIS2. b) Changes in alpha-diversity using Shannon diversity index, analysis was performed by 1- way ANOVA followed by Dunn’s testing for multiple comparisons. Data is presented as box and whisker plot with whiskers extending from 10^th^ to 90^th^ percentile. c) Heatmap of all the significantly differential abundant taxa (aggregated at genus level) in mice evaluated using ANCOM-BC. Color intensity on heatmaps was generated by using z-scores calculated based on relative abundance of each bacterial taxa. d) Time-course graph showing fluctuations in fecal β-defensin-3 values in mice over the course of the experiment. Time points chosen for microbiota analysis are indicated with arrows. e) Composite of mean changes in β-defensin-3 secretion during the three experimental timepoints, presented as box and whisker plot with whiskers extending from 10^th^ to 90^th^ percentile. Statistical differences were calculated using the Kruskal–Wallis test followed by post hoc Dunn’s test for multiple comparisons. f) Correlation between changes in alpha diversity values and defensin secretion in mice during the course of the experiment. Red symbols represent associations between baseline and β-defensin levels, while green and blue symbols represent intervention timepoints and day 21 of recovery time point, respectively. Correlations were generated using Spearman rank index. Baseline days 1 and 7 were combined for the analysis. *n* = 4–5 mice/group. *p* < 0.05 was considered significant. **** = *p* ≤0.0001, *** = *p* ≤0.001, ** = *p* ≤0.01, * = *p* ≤0.05.

The high-fat/high-sugar “Western” diet (HFHSD) altered bacterial composition compared to the baseline (Figure 2a,b) as illustrated by beta and alpha diversity analyses, and these changes normalized within 1 week of discontinuation of the diet. The changes in beta diversity were confirmed by statistical analyses based on Bray Curtis and Aitchison Distance matrices; both matrices revealed similar results (Table 1). While no changes in net bacterial load were found in mice (Supplementary Figure S2c and S2d), changes to the fecal microbial composition were evident at genus level (Figure 2c). The heatmap based on ANCOM-BC analysis revealed that most of the bacterial genera that showed proportional shifts during the exposure to the diet reverted to baseline levels when the diet was discontinued in all mice (Figure 2c). Many bacterial taxa including *Anaerosporobacter, Candidatus Arthromitus*, *Eisenbergiella, Muribaculum, Prevotellaceae*, and *Parasutterella* were lower during the intervention, while taxa like *Anaerotruncus, Angelakisella, Lactococcus*, *Ruminiclostridium*, and *Tyzzerella* were transiently increased. As shown in Figure 2d,e, β-defensin-3 levels decreased during the diet and began to normalize during the recovery period, although they did not return to baseline levels. In addition, fecal β-defensin-3 levels positively correlated with changes in alpha diversity in mice (*r* = 0.6, *p* = 0.002) (Figure 2f). Interestingly, the abundance of bacteria with pro-inflammatory properties, *Candidatus Arthromitus*^43^ (*r* = 0.5, *p* = 0.04) and *Eisenbergiella*^44^ (*r* = 0.6, *p* < 0.001) correlated positively with low defensin levels and Shannon diversity following HFHSD exposure (Table 2).

As in the AMC group, in the HFHSD group, we found small differences between males and females in microbial composition and β-defensin-3 levels. For example, both sexes showed similar trends in alterations in bacterial diversity (Supplementary Figure S1c) as well as proportional shifts in bacterial genera before, during, and following the diet intervention (Supplementary Figure S5). While small differences in bacterial taxa abundances and reduced β-defensin-3 were found in both males and females, the magnitude of this change was greater in females than males, and β-defensin-3 levels normalized only in males post intervention (Supplementary Figure S1d). Specifically, defensin secretion was decreased from baseline levels by > 1-fold in males and > 2-fold in females (Supplementary Figure S1d). This difference might be due to the dissimilarity of microbiota profiles between males and females (Supplementary Figure S1c).

### High salt diet alters fecal microbiota composition and increases fecal β-defensin secretion in mice

**Figure 3.**
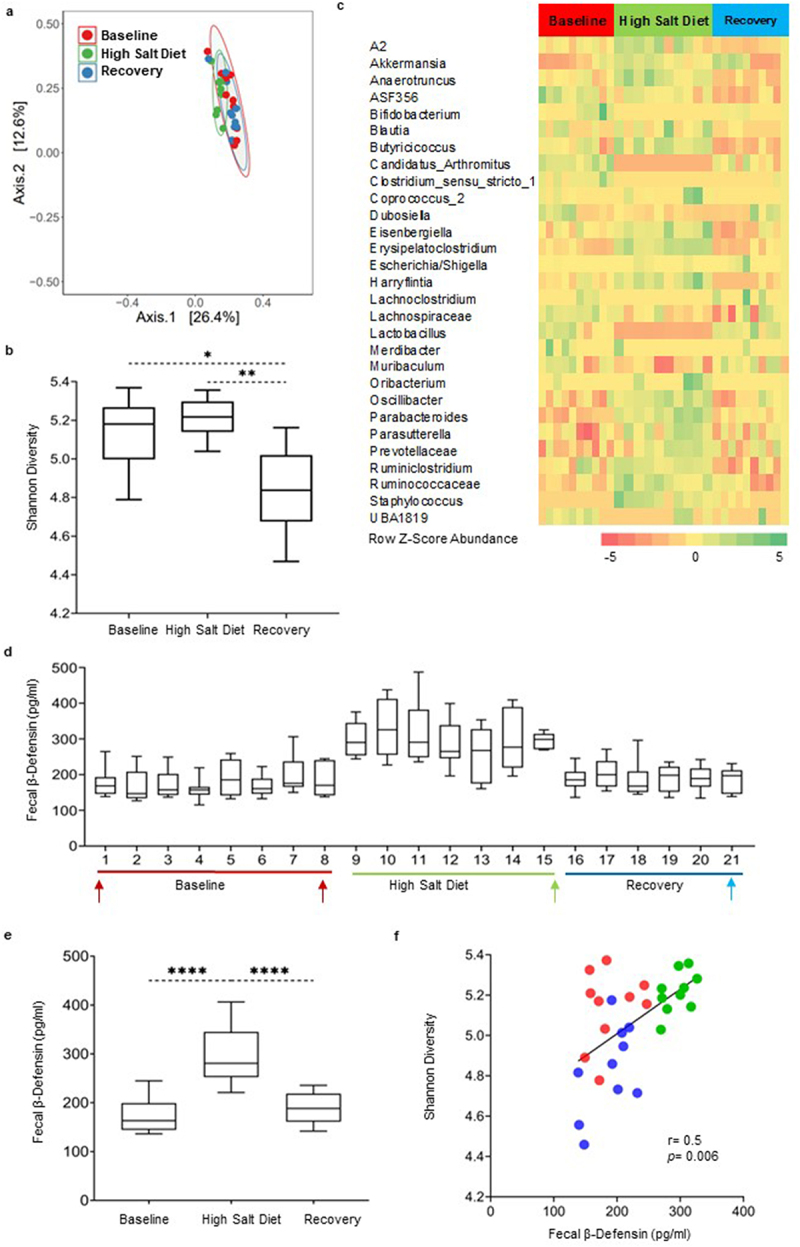
The high salt diet alters gut microbiota composition and modulates host fecal β-defensin secretion in mice. a) PCoA plot based on a Bray Curtis distance matrix analysis of the fecal microbiota composition over the course of the experiment in mice. Community composition was compared by ADONIS2 with 999 permutations. Ellipses indicate 95% confidence intervals and were generated as a distribution around a centroid for each group based on ADONIS2. b) Changes in alpha-diversity using Shannon diversity index, analysis was performed by 1- way ANOVA followed by Dunn’s testing for multiple comparisons. Data is presented as box and whisker plot with whiskers extending from 10^th^ to 90^th^ percentile. c) Heatmap of all the significantly differential abundant taxa (aggregated at genus level) in mice evaluated using ANCOM-BC. Color intensity on heatmaps was generated by using z-scores calculated based on relative abundance of each bacterial taxa. d) Time-course graph showing fluctuations in fecal β-defensin-3 values in mice over the course of the experiment. Time points chosen for microbiota analysis are indicated with arrows. e) Composite of mean changes in β-defensin-3 secretion during the three experimental timepoints, presented as box and whisker plot with whiskers extending from 10^th^ to 90^th^ percentile. Statistical differences were calculated using the Kruskal–Wallis test followed by post hoc Dunn’s test for multiple comparisons. f) Correlation between changes in alpha diversity values and defensin secretion in mice during the course of the experiment. Red symbols represent associations between baseline and β-defensin levels, while green and blue symbols represent intervention timepoints and day 21 of recovery time point, respectively. Correlations were generated using Spearman rank index. Baseline days 1 and 7 were combined for the analysis. *n* = 4–5 mice/group. *p* < 0.05 was considered significant. **** = *p* ≤0.0001, *** = *p* ≤0.001, ** = *p* ≤0.01, * = *p* ≤0.05.

Administration of a high salt diet (HSD) also induced changes in the microbiota composition, as reflected by alpha and beta diversity analyses (Figure 3a,b Table 1). Statistical analyses evaluating both Bray Curtis and Aitchison distance matrices showed a dissimilarity between the intervention and baseline or recovery timepoints as the centroids were distantly grouped. The direction of changes in alpha diversity following a HSD was different from that seen following AMC and HFHSD interventions (Figure 3b). ANCOM-BC analysis of microbial composition revealed a reduced abundance of several microbial taxa including *Lactobacillus* and *Candidatus Arthromitus* (Figure 3c) and increased relative abundance of *Parabacteroides, Akkermansia, Anaerotruncus, Staphylococcus* and *Erysipelatoclostridum*. In contrast to AMC and HFHSD interventions, HSD transiently increased fecal β-defensin-3 secretion up to 2-fold compared to baseline levels in all mice (Figure 3d,e; Supplementary Figure S1f). A positive correlation was found between altered Shannon diversity index values and β-defensin-3 levels (*r* = 0.5; *p* = 0.006) as shown in Figure 3f. Although, there was an overlap between the ellipses representing the three timepoints (Figure 3a and Supplementary Figure S1e), the net compositional change based on p-values in both males and females showed dissimilarity in beta diversity. As shown in Figure 3c, the high salt diet decreased *Lactobacillus* and *Candidatus Arthromitus* relative abundance and increased relative abundance of *Parabacteroides, Akkermansia, Anaerotruncus, Staphylococcus* and *Harryflintia* in both males and females (Supplementary Figure S6).

Interestingly, the abundance of bacteria with pro-inflammatory properties correlated positively with high defensin levels, suggesting that high β-defensin-3 may be due to the presence of these pro-inflammatory bacteria. For example, *Eisenbergiella*^44^ (*r* = 0.5, *p* = 0.02) positively correlated with high defensin levels following HSD exposure, as shown in Table 2. Conversely, bacteria possessing protective/anti-inflammatory properties such as *Clostridium_sensu_stricto_1*^46^ (*r* = −0.46, *p* = 0.04) and *Lactobacillus*^47^ (*r* = −0.7, *p* < 0.001) correlated negatively with β-defensin-3 levels.

### The effect of high salt diet on β-defensin secretion is not mediated by overt inflammation or immune activation

**Figure 4.**
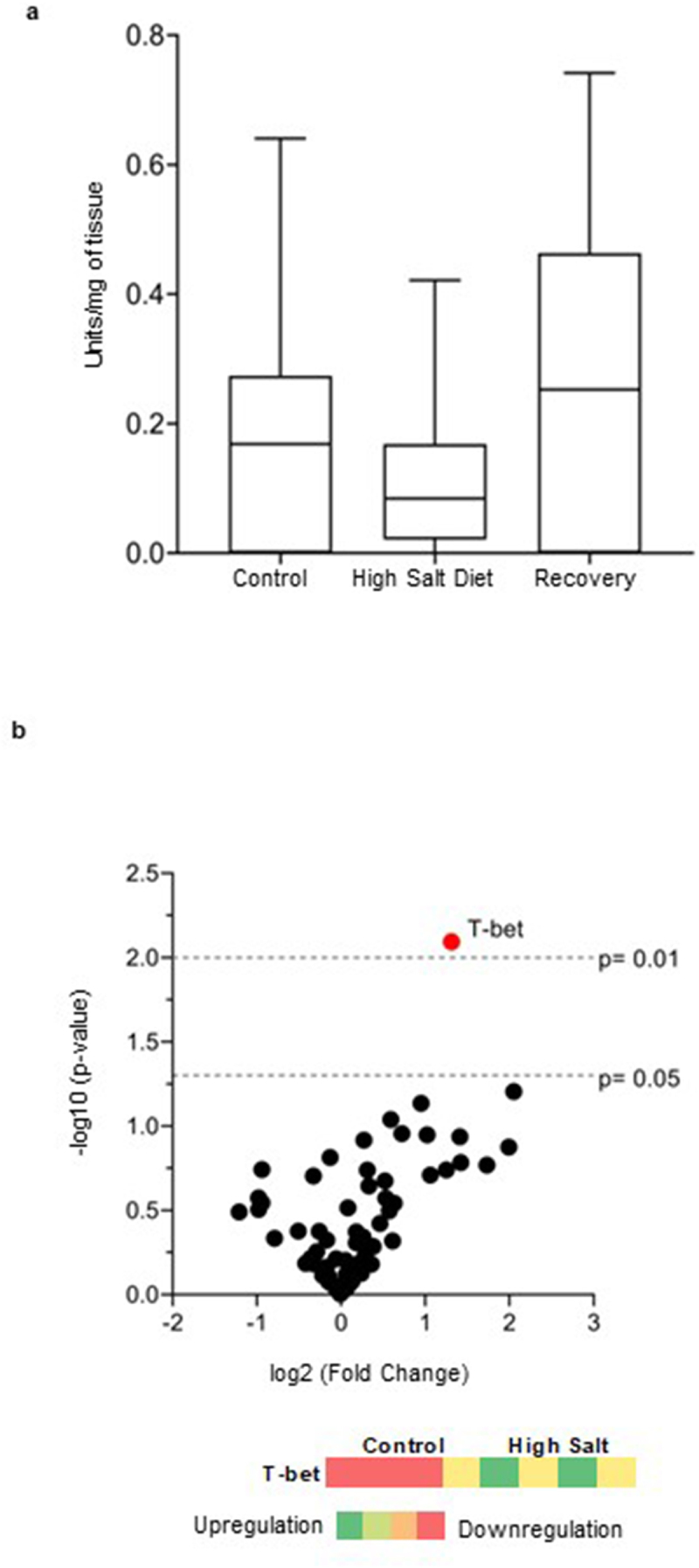
The administration of high salt diet does not induce overt inflammation in mice. a) Colonic myeloperoxidase (MPO) levels in mice who received the control diet, the high salt diet or were given high salt diet followed by 7-day recovery period. Statistical differences between groups were evaluated using 1-way ANOVA (non-parametric) followed by post-hoc Dunn’s analysis. Values are presented as box and whiskers plot with whiskers extending from 10^th^ to 90^th^ percentile, *n* = 5 mice/control and recovery groups; and *n=* 4 mice/treatment group with *p* < 0.05 considered as significant. b) Volcano plot of gene expression in colonic tissues of mice receiving high salt diet or control diet for 1 week. Red dot represents gene with *p* < 0.01. Black dots are genes with 0.01 > *p* > 0.05. Genes present on the right side of the zero were upregulated in high salt diet treated mice but downregulated in control mice. The heat map summarizes the expression of T-bet gene associated with innate immunity in the colon tissues of mice treated with high salt diet or a control diet. Red represents the samples with genes that were downregulated, while green represents the samples with genes that were upregulated. *n* = 4 mice/group. T-bet: T-box transcription factor.

Previous work from our laboratory showed that high salt diet administration for 4 weeks induced a dysbiosis that was associated with an increased susceptibility to colitis in mice.^48^ We questioned whether the increase in β-defensin-3 levels following high salt diet was a component of a diet-induced inflammatory response. We therefore administered a high salt or control diet to mice for one-week and assessed myeloperoxidase (MPO) activity, and inflammation-associated gene expression in the colon. We found no changes in MPO activity in mice following the diet (Figure 4a). As shown in Figure 4b, of the 68 genes related to gut immune and barrier function, T-bet, responsible for regulating intestinal inflammation,^49^ was found to be differentially expressed in control vs. high salt diet treated mice (log2ratio = 1.31; *p* = 0.0081).

### Mild repeated restraint stress does not alter gut microbiota composition or host fecal β-defensin secretion in mice

The effect of mild repeated stress on the microbial composition was negligible, compared to unstressed mice (Supplementary Figure S3a). Similarly, there were no differences in β-defensin-3 secretion in stressed vs. control mice (Supplementary Figure S3b and S3c) or between males and females (Supplementary Figure S3d). No correlations were found between β-defensin-3 levels and overall bacterial diversity (Supplementary Figure S3e).

## Discussion

Our findings indicate that experimentally induced changes in gut microbiota composition result in increases or decreases in β-defensin-3 secretion. Previous studies on β-defensin levels in IBS patients or relevant mouse models were based on samples collected at single timepoints,^33,35,36^ whereas our study involved several time points over 7 days. Given that our interventions produced either increases or decreases in β-defensin-3 compared to baseline, single time point measurements may not capture these changes.

It follows that serial measurements of fecal β-defensins may better capture dysbiosis in IBS patients.^50^ The relationship of innate immune activation, altered microbiota composition and the changes in gut physiology that underlie symptom generation in IBS is complex.^51^ Changes in physiology alter the microbial habitat and thus microbiota composition and, conversely, dysbiosis influences gut physiology, resulting in long-term instability of the host-microbial relationship. Extrinsic factors including antimicrobial exposure or dietary change further influence microbial composition. Based on the results of our study, we propose that variation in β-defensin secretion is secondary to altered microbiota composition and that monitoring β-defensins is a putative biomarker of dysbiosis in IBS. Such a biomarker would help identify those patients in whom microbiota-directed therapies are likely to offer more therapeutic benefit. Current controversies regarding the efficacy of therapies such as probiotics and fecal microbial transfer may simply reflect the fact that while these interventions were aimed at patients exhibiting symptomatic homogeneity, not all patients exhibit,^50^ thereby diluting the overall therapeutic benefit of microbiota-directed therapy.

Previous studies on inducible β-defensins in IBS^33^ and related disorders like Inflammatory Bowel Disease^52,53^ have proposed that defensins are released during a state of active inflammation. Our results show that mice exposed to the high salt diet, previously shown to induce inflammation,^48^ resulted in an increase in fecal β-defensin secretion (Figure 3d,e) and microbial diversity (Figure 3b) without evidence of overt inflammation (Figure 4a,b). This suggests that altered β-defensin-3 secretion was stimulated by altered gut microbiota composition, resulting in an imbalance between commensal protective and proinflammatory bacteria, induced by high salt. Although T-bet was found to be upregulated, its expression was not accompanied by an upregulation of any other genes related to inflammation, for example, IL-17 that has previously been associated with an increased dietary salt.^54,55^ Innate immune activation rather than overt inflammation has been implicated in the pathogenesis of at least a subset of IBS patients, and our results are in line with this in patients with dysbiosis.^33,56^

We recognize that the decreased β-defensin-3 levels following exposure to antimicrobials (Figure 1d,e) and high-fat/high-sugar diet (Figure 2d,e) may appear to be in contradiction to reported single time point elevations in defensin secretion in previous studies.^19,33^ However, these findings are in line with a report of low β-defensin levels in IBS,^35^ and these apparent discrepancies may simply reflect the instability of the microbiota in IBS.^15–17^

Our data indicate that bacterial diversity correlated with β-defensins, suggesting that β-defensin-3 secretion oscillates according to the presence of protective or non-protective (pro-inflammatory) bacteria. A closer inspection of the specific bacterial taxa showed that during antimicrobial exposure bacteria with documented pro-inflammatory effects on the host correlated positively with β-defensin-3 levels. These include bacteria belonging to the genera *Eisenbergiella*,^44^ *Muribaculum*,^45^ and *Prevotellaceae*,^41,42^ (Table 2). Additionally, pro-inflammatory *Candidatus Arthromitus*^43^ was found to positively correlate with β-defensin-3 levels in both AMC and HFSHD groups, while negatively correlated with β-defensin-3 levels in the HSD group. Its decreased abundance could be a consequence of increased defensin secretion during HSD treatment. Interestingly, *Candidatus Arthromitus* is a member of Segmented Filamentous Bacteria (SFB) that have been associated with anti-microbial peptide responses and colonization resistance to pathogenic *Enterobacteriaceae*.^43,57,58^ Conversely, the proinflammatory bacteria, *Eisenbergiella*,^44^ correlated positively with defensin secretion across all groups. This suggests that its low abundance during AMC and HFHSD treatments, and high abundance during HSD treatment, could potentially contribute to the modulation of defensin secretion throughout the treatment periods in all groups. Furthermore, the transcription of inducible defensins such as human β-defensin-2 and -3 has been linked to an increased abundance of species of *Prevotellaceae*.^41,42^ Conversely, negative correlations were seen between defensin levels and the abundance of bacteria with both pro- and anti-inflammatory properties like *Bacteroides*^59,60^ and *Alistipes*^39,40^ (*r* = −0.5, *p* = 0.01) (Table 2). Thus, our findings suggest that homeostatic regulation of defensin secretion in the gut depends on an intricate balance between both protective and pro-inflammatory bacteria (Supplementary Figure S7).

Our proof-of-principle study has limitations. First, the study was performed in healthy mice. Future studies should concentrate on mice that have an IBS-phenotype with disturbed motility and innate immune activation.^19^ This is necessary as a bi-directional relationship exists between the microbiota and host physiology.^61^ Changes in the microbiota alter intestinal permeability,^62^ motility,^63^ and visceral sensitivity,^38^ and conversely, changes, for example, in gut motility, alter microbial composition.^64^ Dietary change, a common factor in IBS symptom expression, together with changes in gut physiology act together to produce temporal instability of the microbiota in at least a subset of IBS patients.^15–17^ We propose that studying β-defensin secretion over time in a humanized mouse model of IBS^19^ in which there is dysbiosis and increased β-defensin-3 would be a logical next step after this proof of concept study and prior to considering clinical studies. A second limitation is the mild nature of the stressor used in this study, as other forms of stress, such as social stress, have been shown to alter microbiota composition and host cytokine levels.^65,66^ Third, we acknowledge the potential of other epithelial constituents and their secretions that could influence the microbial composition of the gut. For instance, antibiotic treatments have been shown to alter the structure of the mucus layer^67^ that impacts the diffusion of antimicrobial peptides and immunoglobulins in the small intestine.^68^ Any alteration in mucus production or composition can contribute to dysbiosis as a result of a breakdown of the gut barrier, hampering intestinal tight junction function and increasing bacterial translocation.^69^ Epithelial cells produce molecules, such as cytokines, and chemokines, which regulate the gut microbiota and protect against pathogens and thereby influence microbial composition. For example, administering antibiotics to treat DSS-induced colitis in mice worsens the condition by decreasing the microbial ligands that activate Toll-like receptors (TLRs) on epithelial cells, which are crucial for promoting the expression of tissue homeostasis and repair mediators, necessary for maintaining healthy colon function.^70^ In addition, a high-fat diet has been shown to cause endoplasmic reticulum stress in intestinal epithelial cells, leading to a reduction in claudin-1 expression and mucus barrier function, which can result in increased serum endotoxin levels and dysbiosis.^71^ The long-term consumption of a high-salt diet has also been associated with altered colonic mucosal immunity, with increased expression of pro-inflammatory genes, and decreased expression of cytokine and chemokine genes in DSS mice.^48^ These studies suggest that elevated levels of pro-inflammatory cytokines and chemokines can influence β-defensin secretion and thus contribute to dysbiosis.

Our results support the concept that changes in fecal β-defensin-3 act as a marker of altered microbiota composition in mice. Defensin comparisons between baseline and relapse may help identify the presence of dysbiosis in chronic GI conditions like IBS and identify patients who may benefit from microbiota-directed therapies.

## Materials and methods

### Study design

Four models of experimentally induced dysbiosis were used to determine changes in fecal β-defensin-3 and to characterize the microbiota composition before, during and after each insult, in both males and females. We used (1) an antimicrobial cocktail (AMC) in drinking water, (2) a high-fat/high-sugar diet (HFHSD), (3) a high salt diet (HSD), and (4) mild repeated restraint stress (MRS). In the AMC and dietary studies, we employed a 1-week intervention period which was preceded by a week-long baseline and followed by a recovery period. The same group of mice were followed over the course of 3-week period, and fecal samples were collected every day to measure the fluctuations in each β-defensin-3 (a murine homologue of human β-defensin-2) levels and microbiota. In MRS studies, mice comparisons were made between a control and a stressed group. Fecal samples were collected for consecutively 8 days.

### Animals

All experiments were performed using 7- to 10-week-old specific pathogen-free (SPF) C57BL/6 mice except studies using MRS that were performed in NIH Swiss SPF mice. All mice were purchased from Taconic Biosciences and were housed in plastic cages at McMaster University’s central animal facility (CAF) under a 12 h light/dark schedule in a temperature-controlled vivarium with *ad libitum* access to food and water. Mice received irradiated control diet (1020, Teklad, with 20% of calories from protein, 29% from fat, and 51% from carbohydrates, containing 0.4% Na and 0.7% Cl) and sterile tap water ad libitum upon arrival as well as during baseline and recovery periods. Experiments started 1-week after arrival in the CAF to allow the animals to habituate to the novel environment before initiating the experimental protocols. Cages were changed before and after the initiation of the experiments. The status of the animals was monitored by daily general examination and body weight measurements. Food and water intake was also measured at alternate days. All mice were handled only in a level II biosafety hood to prevent bacterial contamination. Mice were sacrificed using a standardized protocol under Isoflurane (Fresenius Kabi Canada, Toronto, ON) anesthesia. The experiments were approved by the McMaster University animal ethics committee under the Animal Utilization Protocol #18-08-35.

### Antimicrobials intervention

The AMC was administered following a previously established protocol.^37,38^ Briefly, C57BL/6 males (*n* = 5) and females (*n* = 5) received a mixture of non-absorbable antimicrobials (neomycin 5 mg/mL, bacitracin 5 mg/mL, and pimaricin 1.25 microg/mL) in their drinking water for 1 week. For the preparation of antimicrobials mixture, neomycin and bacitracin were weighted and stirred in water for 30 min to fully dissolve the powdered antimicrobials before adjusting the pH. At the end, pimaricin was added to the mixture. The antimicrobials were prepared fresh every 48 h.

### Dietary interventions

Two different diets were used for our dietary intervention experiments: a high-fat/high-sugar diet (HFHSD) and a high salt diet (HSD). The protocol consisted of two separate groups of mice (*n* = 5 males and 5 females each) that were fed either HFHSD or HSD for 1 week. The HFHSD was composed of Kcal%: Carb, 35; Fat, 45; Fiber, 0.0 (Prot, 20, # D12451, Research Diets, New Brunswick, NJ), and the HSD was the same as the control diet administered during the baseline period but supplemented with 4% NaCl (TD.130834) ordered from Envigo, Teklad, WI USA. Both groups were administered regular sterile drinking water during the intervention periods.

### Mild repeated restraint stress

Both male and female mice were divided into two groups consisting of a control group (*n* = 4 males and 4 females) and stress group (*n* = 3 males and 4 females). The mice from the stress group were placed in a plexiglass restrainer for 30 min daily, at a fixed time of the day, for 8 days. The restrained mice were placed in a new cage and were positioned in the restrainers in a way that allowed minimal to no movement but a continued supply of oxygen. Following the stress cycle, mice were transferred back to their home cage and were left undisturbed for the rest of the day. Fecal pellets were collected from both groups every day for the course of the experiment. NIH Swiss mice were used as they have been previously shown to be more sensitive to stress than other mouse strains.^72^

### Assessment of overt inflammation using myeloperoxidase (MPO) activity following HSD

A separate group of mice (*n* = 5 males and 5 females/timepoint; total mice = 30) followed the same HSD protocol as mentioned above but were sacrificed at the end of every timepoint (baseline, intervention, and recovery). After sacrifice, the entire colon was removed, and tissue samples were snap-frozen in liquid nitrogen for assessment of MPO activity as described previously.^73^ The MPO activity was expressed in units/milligrams of tissue. Outliers were removed wherever appropriate.

### Mouse β-defensin-3 Enzyme Linked Immuno-Sorbent Assay (ELISA)

Mouse β-defensin-3 were measured by ELISA (MyBioSource, MBS034940, San Diego, CA, US) according to manufacturer’s instructions. Briefly, each sample was separately weighted, and PBS was added accordingly (10 µl/mg of sample). Then, samples were mechanically homogenized with 3.0 mm ceramic beads (Sigma, USA) in a bullet homogenizer (Next Advance, NY USA). The samples were homogenized for 10 min at maximum speed, followed by centrifugation at 3000 rpm for 20 min. The supernatants were then collected and loaded on the ELISA plate with blanks and standards. Each sample was run in duplicate. Optical density was read at 450 nm using ELx808 absorbance reader (BioTek, Winooski, VT, USA), and the results were expressed in pg/ml.

### DNA isolation and 16S rRNA illumina sequencing

Fecal samples were chosen for Illumina sequencing of the16S rRNA gene, at baseline, day 1 and day 7, during intervention at day 15 and at the end of recovery period at day 21. Fecal samples were homogenized and processed to extract DNA based on previously described protocols.^19,48^ Polymerase chain reaction (PCR) amplification of the hypervariable 3 (V3) region of the 16S rRNA gene was performed as previously described.^74,75^ Purified PCR products were sequenced using the Illumina MiSeq platform at the McMaster Genomics facility. Isolated gDNA saved from the Illumina sequencing protocol was used to quantify bacterial load using RT-PCR. Briefly, gDNA was collected using one fecal pellet per time point. Amplifications were performed on Step one plus real-time PCR system (BioRad, ON, CA) (95°C for 10 min, followed by 40 cycles of 95°C for 15 s, 55°C for 40 s and 62°C for 60 s) using SYBR Green-based PCR master mix (Applied Biosystems) and the following primers 926 F (5’ AAA CTC AAA KGA ATT GAC GG) at *E. coli* position 908–926 and 1062 F (5’ CTC ACR RCA CGA GCT GAC) at *E. coli* position 1081–1064 targeting regions V6-V8, as described previously.^76^ Samples were run in duplicates, and data were normalized with reference to baseline (day 1 and day 7) timepoint measurements and analyzed using the two-ΔCt method. A melting analysis was also carried out following the last cycle of each amplification to confirm the amplification specificity. For each experimental group (AMC, HFHSD, HSD), a standard curve with efficiency between 91% and 100% was run together with the samples, as stated earlier.^19^

### 16S rRNA processing and analysis/16S Ribosomal RNA gene analysis

All sequences obtained following sequencing were processed using Cutadapt to trim and filter adapter sequences and PCR primers from the raw reads with a minimum quality score of 30 and minimum read length of 100 base pairs, as previously described.^19^ Further, denoising Algorithm 2 (DADA 2) package in R was used to align the paired-end sequences and perform all the subsequent quality filtering and removing chimeras.^77^ Taxonomy was assigned using the Silva reference database.^78^ ASVs not assigned to bacterial genera or assigned to either mitochondria or chloroplast were removed from analysis. Any ASVs identified as chimeric by de novo detection in DADA2 R package were removed. Mouse samples had an average of 69,846.46 reads per sample after filtering, ranging from 11,055 to 171,477 reads per sample. The analysis was performed by merging all Illumina runs, running all analyses together and then sub-setting the samples according to each experimental group used in the study when required. Phyloseq and vegan packages for R, version 4.1.1 (R foundation for Statistical Computing) were used for statistical analyses. Following the filtering of non-bacterial taxa from samples, principal coordinate analysis (PCoA) was performed using the Bray-Curtis and Aitchison Distance matrices to evaluate beta-diversity. Statistical differences in beta-diversity between the experimental groups were analyzed using the ADONIS2 function in the vegan package. Reads from baseline and days 1 and 7 were combined and considered as a single baseline timepoint. To evaluate differences in alpha diversity, Shannon diversity was employed in the absence of rarefaction. Differences in alpha diversity were assessed using one-way ANOVA with FDR correction for multiple comparisons using Dunn’s test. The identification of any differentially abundant bacterial taxa associated with baseline, intervention and recovery periods was conducted using the ANCOM-BC package,^79^ after filtering the dataset to remove any ASVs with a frequency of less than 4 in at least 10% of the samples and converting the absolute abundance to relative abundance. The samples were then aggregated at the genus level, merging bacteria belonging to the same strain into a single entity. Statistical significance on differentially abundant taxa between groups was corrected for multiple comparisons using false discovery rate (FDR). False discovery rate with a Q value of 0.05 was used for the multiple testing of bacterial taxa. Color intensity on heatmaps was generated by using z-scores calculated relatively within each data set (bacteria). PCoA plots, heatmaps and diversity box plots were plotted using phyloseq, vegan and ggplot packages for R or using GraphPad Prism version 9.

### Gene expression analysis

RNeasy Mini Kit (Qiagen, Germany) was used to extract and purify total RNA from whole colonic tissue samples according to manufacturer’s instructions. Additional steps were performed during purification to remove DNA traces from the samples using the RNease-Free DNase Set (Qiagen). A custom Code Set panel that included 68 genes related to immune, gut barrier, and neurobiology functions was run according to the manufacturer’s instructions (NanoString Technologies Inc., Seattle, WA, USA), and data were analyzed with nSolver 4.0 software (Nanostring Technologies Inc.). The log2 ratios built from the data obtained were then plotted as volcano plots and represented as heatmaps.

### Data analysis

Statistical analysis was carried out using GraphPad Prism 9 (GraphPad Software, La Jolla, CA, USA), and R (version 4.1.1). Statistical comparisons were performed using one-way ANOVA, two-way ANOVA, Kruskal−Wallis, Mann−Whitney or unpaired *t*-test, as appropriate. Dunn’s test or Benjamini, Krieger and Yekutieli were used, when performing post hoc tests for multiple comparisons. A non-parametric Spearman rank test followed by FDR corrections was used when performing correlations. Two-tailed statistical tests were used. A p-value of less than 0.05 was considered to be statistically significant.

## Supplementary Material

Supplemental Material

## Data Availability

The raw 16S rRNA sequences and the gene expression data have been deposited at NCBI under the umbrella BioProject PRJNA935320. Individual public projects under this BioProject are SRA BioProject (PRJNA932045) and Geo BioProject (PRJNA934868). Supplemental data that supports the findings of this study can be accessed online at https://doi.org/10.6084/m9.figshare.23116457.v1.
