# Supplementary material for: Alterations in fecal β-defensin-3 secretion as a marker of instability of the gut microbiota
Source: Gut Microbes. 2023 Jul 18;15(1):2233679. doi: 10.1080/19490976.2023.2233679 (PMC10355691; doi:10.1080/19490976.2023.2233679)
Supplement: Supplemental Material [file KGMI_A_2233679_SM1292.zip › Supplemental material/Supplemental material legends.docx]

*Supplemental online material*

**Alterations in fecal β-Defensin-3 secretion as a marker of instability of the gut microbiota**

**Zarwa Saqib^1^,** Giada De Palma^1^, Michael Surette^1^, Premysl Bercik^1^, and Stephen Michael Collins^1*^

Farncombe Family Digestive Health Research Institute, Department of Medicine, Faculty of Health Sciences, McMaster University, Hamilton, ON, Canada

***Corresponding Author:**

Dr. Stephen Collins

Email: [scollins@mcmaster.ca](mailto:scollins@mcmaster.ca) scollins@mcmaster.ca

**Supplementary Figure 1. The nature of changes in net bacterial composition and β-defensin-3 levels in male and female mice is dependent on the process underlying the induction of dysbiosis.** a) PCoA plot based on a Bray Curtis distance matrix analysis of the fecal microbiota composition on specific days over the course of the experiment in AMC treated males and females. b) Composite of mean changes in β-defensin-3 secretion during the 3 experimental timepoints in AMC treated male and female mice. Values are presented as box-whisker plot with the median in the centre line and whiskers extending from 10^th^ -90^th^ percentile. c) PCoA plot based on a Bray Curtis distance matrix analysis of the fecal microbiota composition on specific days over the course of the experiment in HFHSD treated males and females. d) Composite of mean changes in β-defensin-3 secretion during the 3 experimental timepoints in HFHSD treated male and female mice. e) PCoA plot based on a Bray Curtis distance matrix analysis of the fecal microbiota composition on specific days over the course of the experiment in HSD treated males and females. f) Composite of mean changes in β-defensin-3 secretion during the 3 experimental timepoints in HSD treated male and female mice. Community composition based on each intervention used was compared by ADONIS2 with 999 permutations. Each dot in each intervention timepoint on PCoA plot represents the bacterial microbiota of an individual mouse. Statistical differences in β-defensin levels were calculated using 2-way ANOVA (non-parametric) followed by FDR testing for multiple comparisons. *n* = 4-5 mice/group. *p* < 0.05 was considered significant. Males are represented by triangles and females by circles.

**Supplementary Figure 2. No changes in bacterial load were associated with interventions in all experimental groups.** a) Changes in bacterial load before, during and after AMC intervention in male and female mice. b) Changes in bacterial load before, during and after HFHSD in male and female mice. c) Changes in bacterial load before, during and after HSD in male and female mice. Data was normalized in reference to baseline days 1 and 7 measurements. Statistical differences between groups were evaluated using 1-way ANOVA (non-parametric) followed by Dunn’s test for both males and females. Values are presented as box-whisker plot with the median in the center line and whiskers extending from 10-90^th^ percentile; *n* = 5 mice/group with *p* < 0.05 considered significant.

**Supplementary Figure 3. Mild repeated restraint stress does not alter microbiota composition or modulate fecal β-defensin-3 secretion in mice.** a) Changes in bacterial diversity following mild repeated restraint stress (stress) or no stress (control) as evaluated using the Shannon diversity index. b) Time-course graph showing fluctuations in fecal β-defensin-3 values in mice over the course of the experiment. Time points chosen for microbiota analysis are shown with arrows. c) Composite of mean changes in β-defensin-3 secretion in both control and stress mice. Data is presented as median with interquartile range and whiskers extending from 10^th^ to 90^th^ percentile. Statistical differences in mice β-defensin-3 levels were calculated using unpaired, two-tailed T test followed by Mann-Whitney statistical analysis. d) Composite of mean changes in β-defensin-3 secretion in both control and stress mice based on sex. Data is presented as median with interquartile range and whiskers extending from 10^th^ to 90^th^ percentile. Statistical differences in mice β-defensin-3 levels were calculated using one-way ANOVA. e) Correlation between changes in alpha diversity values and defensins secretion in mice during the course of the experiment. Red symbols represent associations between stress mice alpha diversity and β-defensin-3 levels, while black symbols represent associations between control mice alpha diversity values and β-defensin-3 levels. Correlations were generated using Spearman rank index. *p* < 0.05 was considered significant. Control group: *n= 4* males; *n= 4* females. Mild Restraint Stress group: *n= 3* males and *n= 4* females.

**Supplementary Figure 4. Differentially abundant bacterial taxa (genus) in male and female mice before, during and following antimicrobial cocktail intervention**. Heatmap of all the differentially abundant taxa (aggregated at genus level) in male and female mice evaluated using ANCOM-BC. Prior to taxonomic analyses, the dataset was filtered to eliminate ASVs with low read counts following which data was transformed to relative abundance and then aggregated at the genus level. Statistical significance on differentially abundant taxa between groups was corrected for multiple comparisons using FDR (*q* <0.05). Color intensity on heatmaps was generated by using z-scores calculated based on relative abundance of each bacterial taxa. B represents Baseline; T represents Intervention and R represents Recovery.

**Supplementary Figure 5. Differentially abundant bacterial taxa (genus) in male and female mice before, during and following high fat/ high sugar diet intervention**. Heatmap of all the differentially abundant taxa (aggregated at genus level) in male and female mice evaluated using ANCOM-BC. Prior to taxonomic analyses, the dataset was filtered to eliminate ASVs with low read counts following which data was transformed to relative abundance and then aggregated at the genus level. Statistical significance on differentially abundant taxa between groups was corrected for multiple comparisons using FDR (*q* <0.05). Color intensity on heatmaps was generated by using z-scores calculated based on relative abundance of each bacterial taxa. B represents Baseline; T represents Intervention and R represents Recovery.

S**upplementary Figure 6. Differentially abundant bacterial taxa (genus) in male and female mice before, during and following high salt diet intervention**. Heatmap of all the differentially abundant taxa (aggregated at genus level) in male and female mice evaluated using ANCOM-BC. Prior to taxonomic analyses, the dataset was filtered to eliminate ASVs with low read counts following which data was transformed to relative abundance and then aggregated at the genus level. Statistical significance on differentially abundant taxa between groups was corrected for multiple comparisons using FDR (*q* <0.05). Color intensity on heatmaps was generated by using z-scores calculated based on relative abundance of each bacterial taxa. B represents Baseline; T represents Intervention and R represents Recovery.

**Supplementary Figure 7. Taxonomic differences in the fecal microbiota among all the animal models.**Bar plots showing the relative abundance and distribution of the top 5 most abundant microbial phyla specified at genus level present in the individual timepoints. The less abundant phyla are grouped under the “Other” category; shown in grey. For simplification, the less abundant genus of each top 5 phyla is grouped as “Other”. *n= 10* mice/ intervention group (AMC, HFHSD, HSD); and *n= 8* mice/ control group and *n= 7* mice/ stress group.

**Supplementary Table 1.** p and R^2^ (group- variance) values based on the ADONIS2 statistical analysis of the Bray Curtis- distance metric (top) and Aitchison distance metric (bottom) on the effect of antimicrobials, high-fat/high sugar diet and high salt diet on fecal microbiota composition in male and female mice.
