# Supplementary figures and images for: Alterations in fecal β-defensin-3 secretion as a marker of instability of the gut microbiota
Source: Gut Microbes. 2023 Jul 18;15(1):2233679. doi: 10.1080/19490976.2023.2233679 (PMC10355691; doi:10.1080/19490976.2023.2233679)

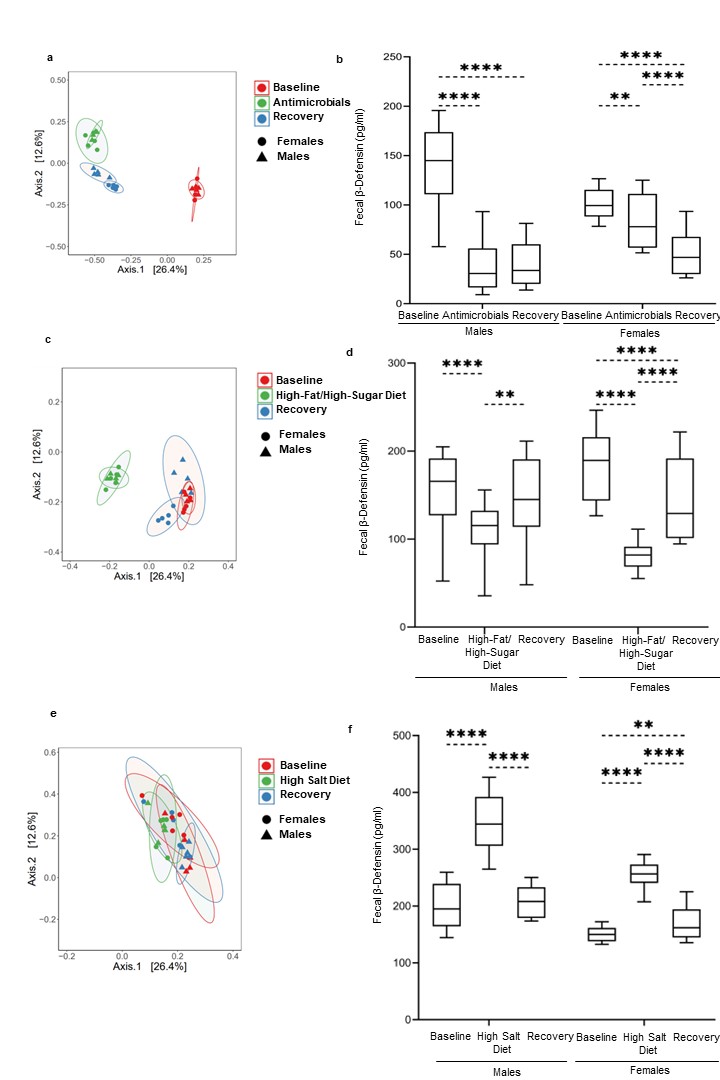

Supplement: Supplemental Material [file KGMI_A_2233679_SM1292.zip › Supplemental material/Supplementary Figure 1.JPG]

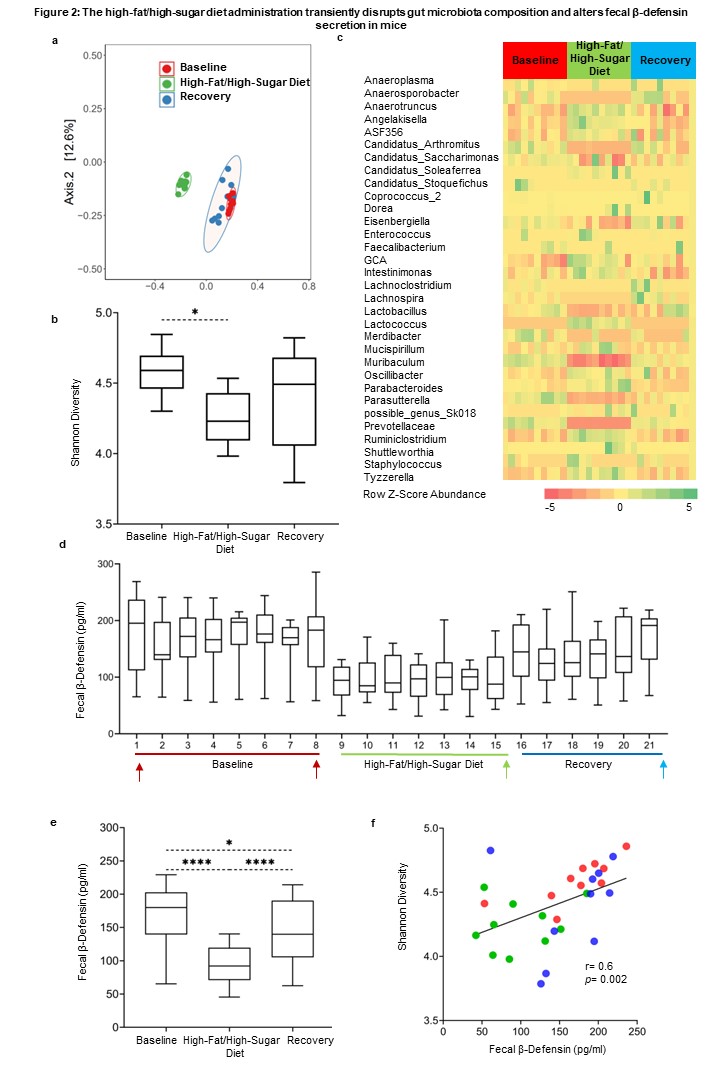

Supplement: Supplemental Material [file KGMI_A_2233679_SM1292.zip › Supplemental material/Supplementary Figure 2.JPG]

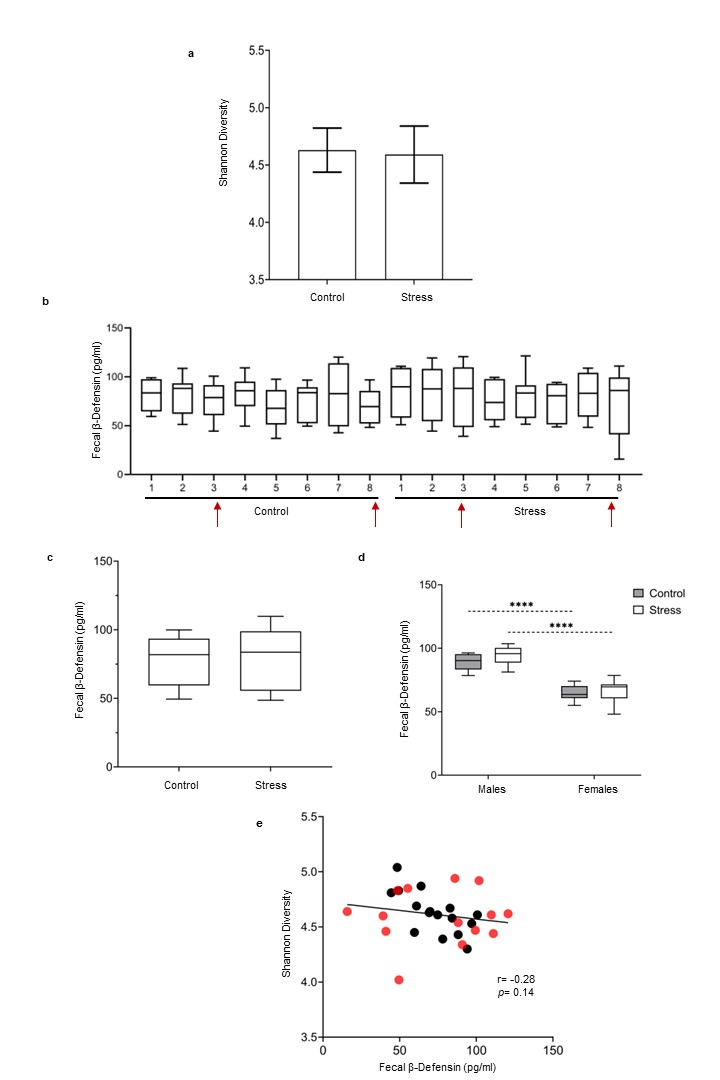

Supplement: Supplemental Material [file KGMI_A_2233679_SM1292.zip › Supplemental material/Supplementary Figure 3.JPG]

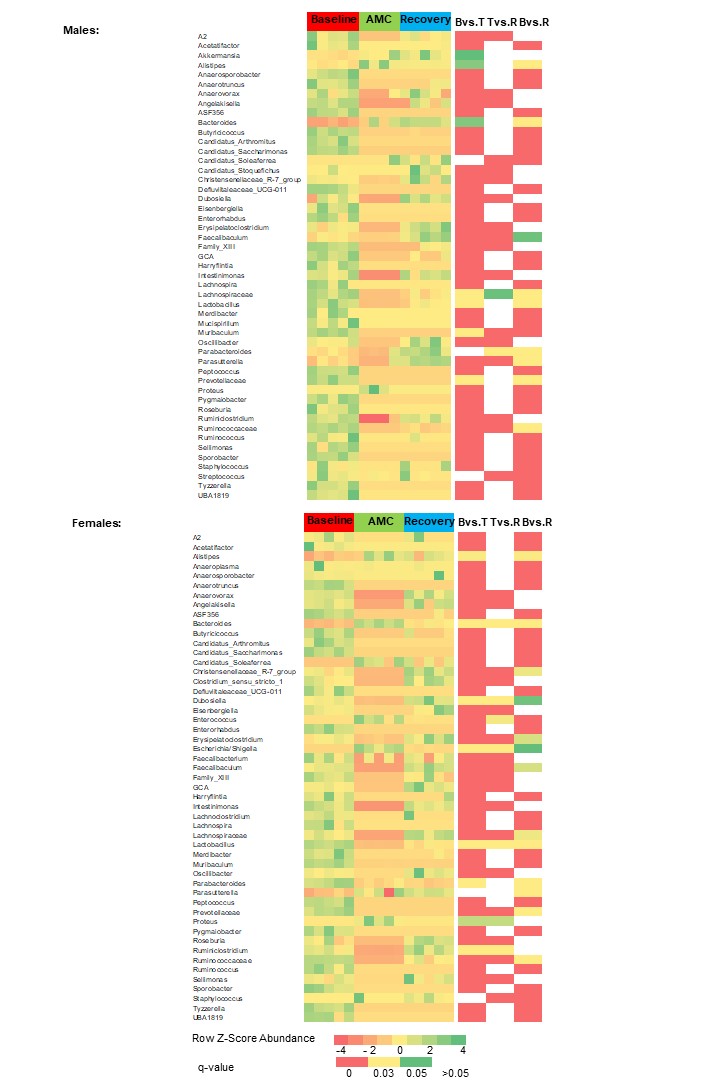

Supplement: Supplemental Material [file KGMI_A_2233679_SM1292.zip › Supplemental material/Supplementary Figure 4.JPG]

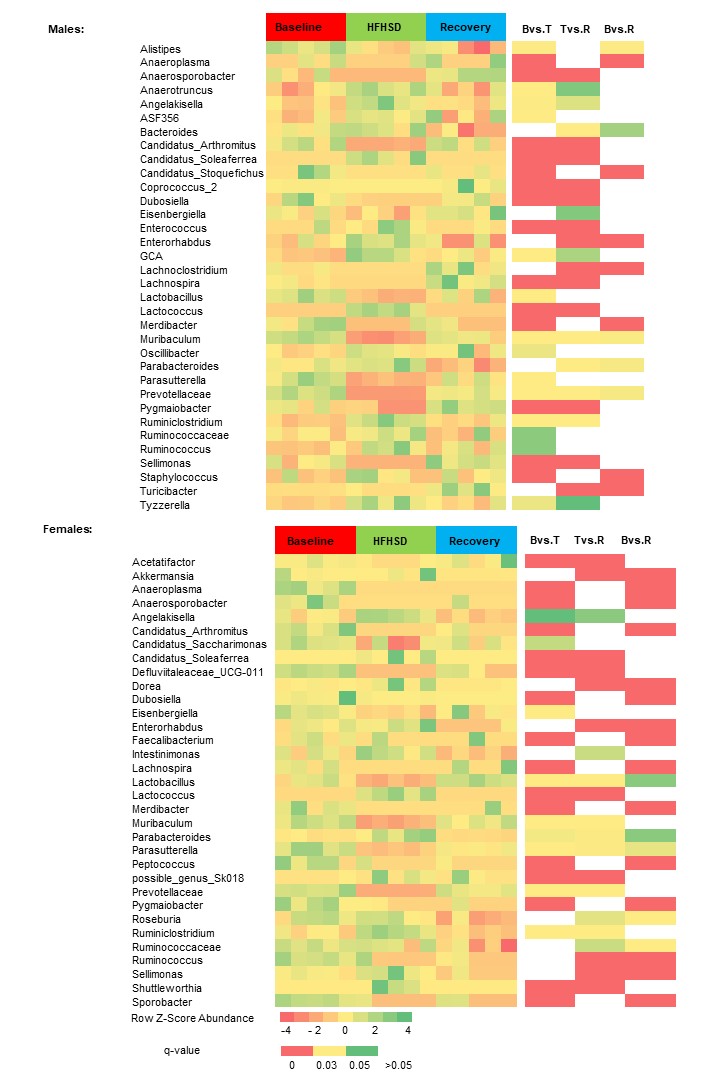

Supplement: Supplemental Material [file KGMI_A_2233679_SM1292.zip › Supplemental material/Supplementary Figure 5.jpg]

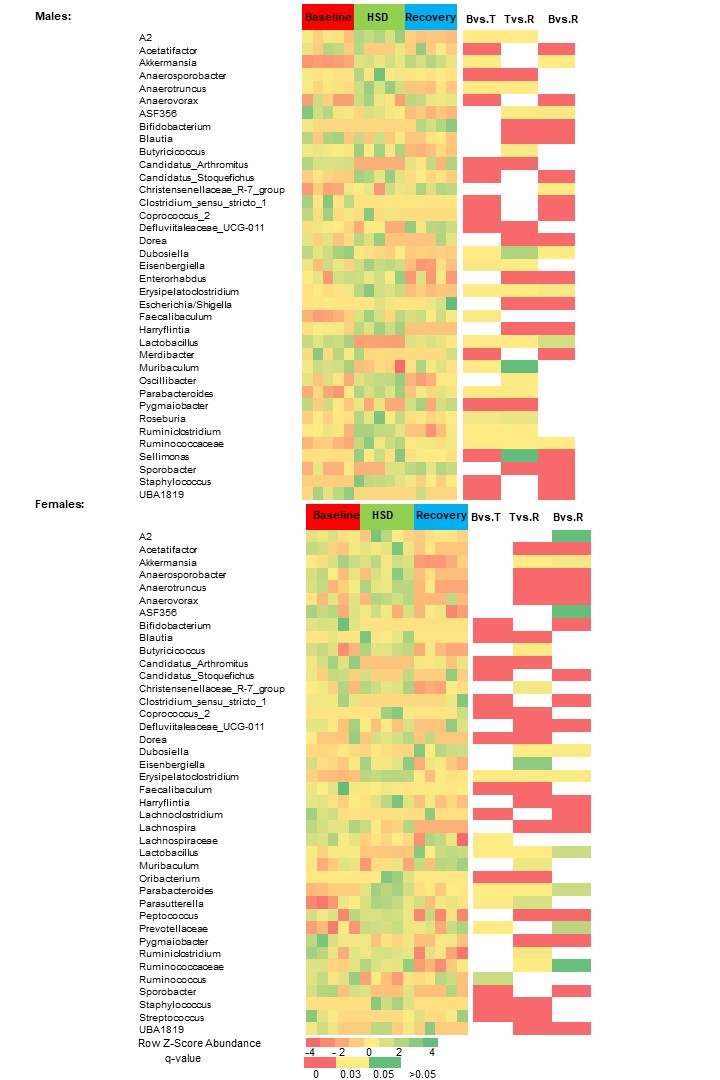

Supplement: Supplemental Material [file KGMI_A_2233679_SM1292.zip › Supplemental material/Supplementary Figure 6.jpg]

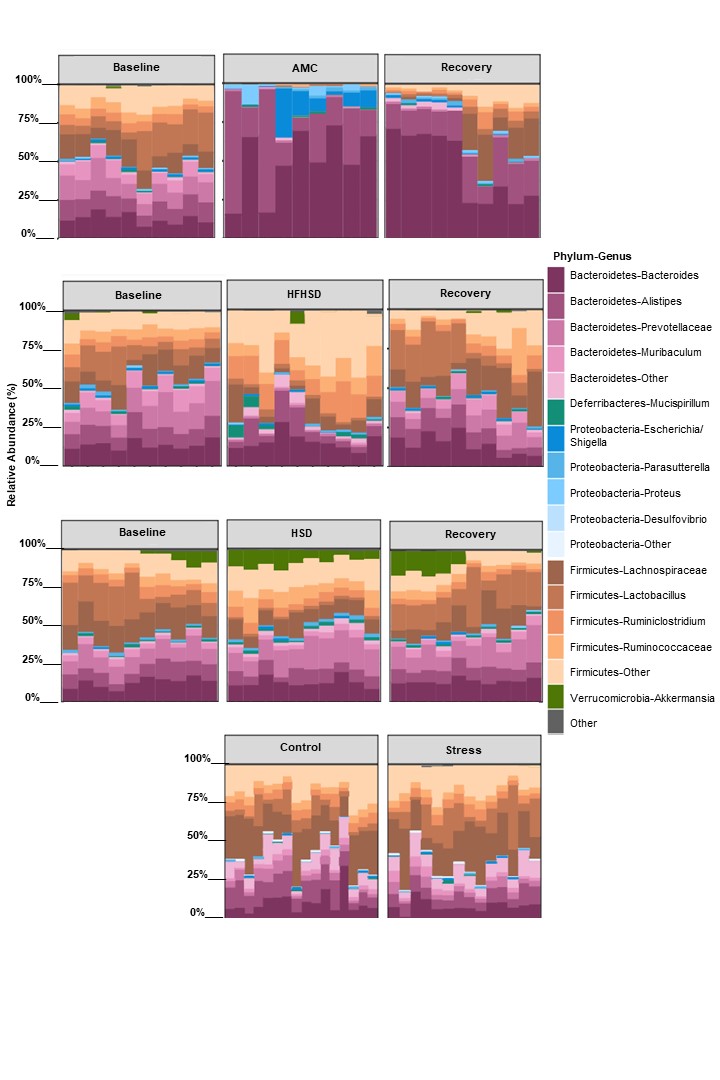

Supplement: Supplemental Material [file KGMI_A_2233679_SM1292.zip › Supplemental material/Supplementary Figure 7.JPG]

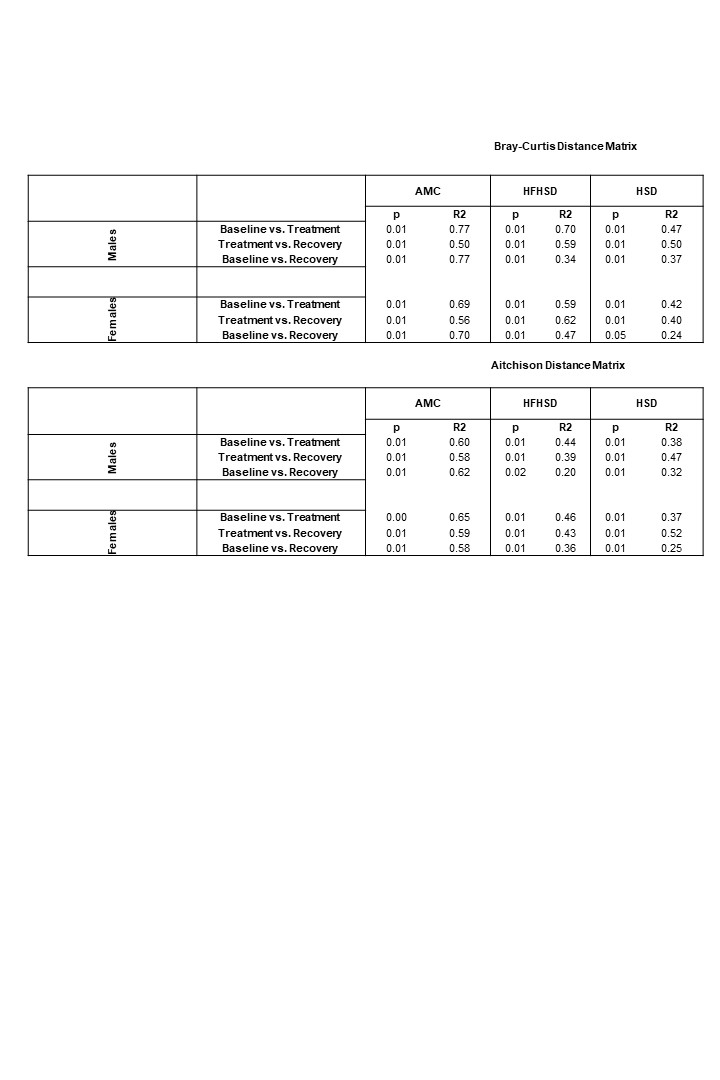

Supplement: Supplemental Material [file KGMI_A_2233679_SM1292.zip › Supplemental material/Supplementary Table 1.JPG]
